# Keratocystic odontogenic tumor: A biopsy service’s experience with 104 solitary, multiple and recurrent lesions

**DOI:** 10.4317/medoral.21181

**Published:** 2016-07-31

**Authors:** Ibrahim-Olajide Bello

**Affiliations:** 1 Assistant Professor. Department of Oral Medicine and Diagnostic Sciences, College of Dentistry, King Saud University, Saudi Arabia

## Abstract

**Background:**

Keratocystic odontogenic tumor (KCOT) is a clinically significant cystic lesion of odontogenic origin. This study aimed to retrospectively review and describe the clinicopathologic features of KCOT and to objectively compare the clinical and histological features of solitary, multiple and recurrent KCOT in a Saudi Arabian population.

**Material and Methods:**

Biopsy request forms, pathology records and archival materials (all histological slides) of 104 cases of KCOT from 75 patients were retrieved. Demographic and clinical details as well as histological evaluation were analyzed and compared between the 3 groups using chi-squared or Mann-Whitney tests of association as appropriate.

**Results:**

Significant differences were noted in the age of presentation, location and association with impaction between multiple and solitary cases. Histologically, there was a difference in the mitotic count, presence of satellite cysts and proliferating odontogenic epithelium between solitary and multiple lesions. There was no difference between the KCOT that later recurred and solitary lesion which did not recur even when matched clinically for age, sex and location. There were differences when solitary KCOT that later recurred or recurrent KCOT were compared with multiple lesions. Multiple lesions still had more significant proliferative activity parameters than solitary recurrence-related KCOT.

**Conclusions:**

KCOTs in Saudi Arabians are not different from those reported from other parts of the world. Clinical and histological analyses showed multiple KCOT is different from its solitary recurrent or non-recurrent counterparts and has a higher proliferative activity than both. Clinicohistologic features alone cannot wholly explain the behavior of KCOT.

**Key words:**Descriptive study, keratocystic odontogenic tumor, odontogenic keratocyst, solitary, multiple, recurrent.

## Introduction

The odontogenic keratocyst, currently known as keratocystic odontogenic tumor (KCOT) is a benign cystic lesion of odontogenic origin with a potentially aggressive and infiltrative behavior. It has a characteristic lining of parakeratinized stratified squamous epithelium and could present radiographically as unilocular or multilocular radiolucency (1,2). KCOT frequently occurs as a single lesion, although some patients present with multiple synchronous or metachronous lesions. Such cases are often attributable to nevoid basal cell carcinoma syndrome (NBCCS) or Gorlin syndrome (3). Previous studies that have attempted to compare solitary and multiple cases of KCOT have concluded that there are clinical, histological and histometrical differences particularly with NBCCS-related cases (4,5).

KCOT is well known for its relatively higher recurrence rates in comparison to odontogenic cysts (6). Majority of studies have rightly focused on the surgical management in relation to recurrence of the lesion (7,8). Despite the huge volume of literature, there is currently no high level evidence to support the efficacy of the various treatment interventions for KCOT (9). Biopsy service-based comparison of recurrent and non-recurrent KCOT has been previously carried out (10). The findings seemed to suggest that recurrence is mainly due to operative factors rather than clinical and histological differences.

There is no detailed information on the clinical and histological features of patients with KCOT in Saudi Arabian patients. A recent study on odontogenic tumors briefly mentioned some of its demographic features (11). This present study aimed at achieving two goals: firstly, to retrospectively review and describe the general clinicopathologic features of the KCOT cases, and secondly, to objectively compare these features between solitary, multiple and recurrent cases of KCOT in our service. In both instances, the findings were related to those already documented in the literature.

## Material and Methods

The registration and ethical approval of this study were given by the College of Dentistry Research Center (number FR 0279). All patients had previously given a signed informed consent in their case files regarding inclusion into epidemiological and research work on the tissues obtained from them for diagnostic purposes. The materials used in this study were collected from the pathology records and biopsy request forms of the histopathology laboratory of the College of Dentistry, King Saud University, Riyadh, Saudi Arabia. They comprised cases from patients seen between 1984 and 2014. Cases were included if diagnosed at this laboratory; if the original slides or histological blocks are still available in the archives, and on evaluation meet the histologic definition of KCOT (1,2).

The slides chosen for evaluation were preferentially those produced from the definitive treatment of the patients, except in cases where the only the incisional biopsy diagnostic slides were available or better represented the KCOT. The histological blocks were retrieved and new slides made for evaluation in cases where the original slides were missing. Of 127 cases collected (including newly made slides), 23 cases were excluded because they were orthokeratinized odontogenic cysts (n =11) or unicystic ameloblastoma or other cysts (n=12).

In all, 104 cases from 75 patients were finally available for this study. Single lesions were found in 65 patients, 30 cases of multiple lesions were obtained from 10 patients in addition to 9 recurrent cases from patients with solitary lesions. Recurrences from multiple cases were excluded because of difficulty in verifying if they were true recurrences or new cases in some patients. All slides were reevaluated by the author.

Data collected from the records included the age and gender of the patients, location of the lesion, presenting complaint and association with tooth impaction. Information on recurrence as well as whether lesions were single or multiple lesions in individual patients were also collected. Recorded treatments indicated that apart from two lesions that were masurpialized and one resected, other lesions were enucleated with additional use of chemical cauterization (Carnoyl solution) and peripheral ostectomy or curettage in some cases. Since the exact details of the operation were not included in our pathology records or request forms, it was considered prudent not to explore this further. Similarly, radiographs were no longer available in many cases thereby necessitating the exclusion of radiographic details from analyses.

The data from the histologic characteristics evaluated from the slides was qualitatively divided into two groups thus:

I) Mitotic count was analyzed by scanning the epithelial lining for unequivocal mitosis above the basal layer in 10 random fields at 40 x magnifications and the total count recorded. Basal layer mitoses were disregarded. Three or more mitotic figures were considered significant.

II) Inflammation was considered significant if it altered the epithelial lining of the lesion from classic to that seen in inflammatory odontogenic cysts and/or if it was densely covering at least a quarter of the cyst wall.

III) All other histological variables evaluated were scored as ‘present’ or ‘absent’ without using the quantity or number for analysis.

Quantitative data was analyzed using percentages, means and ranges and if non-parametric, medians calculated and associations tested by Mann-Whitney test. Qualitative data was similarly tested using Pearson chi-squared, or Fisher’s exact tests depending on analyses. Significance was set at *P* ≤ 0.05 (2 sided).

## Results

- General Patient Profile

**Figure 1:**
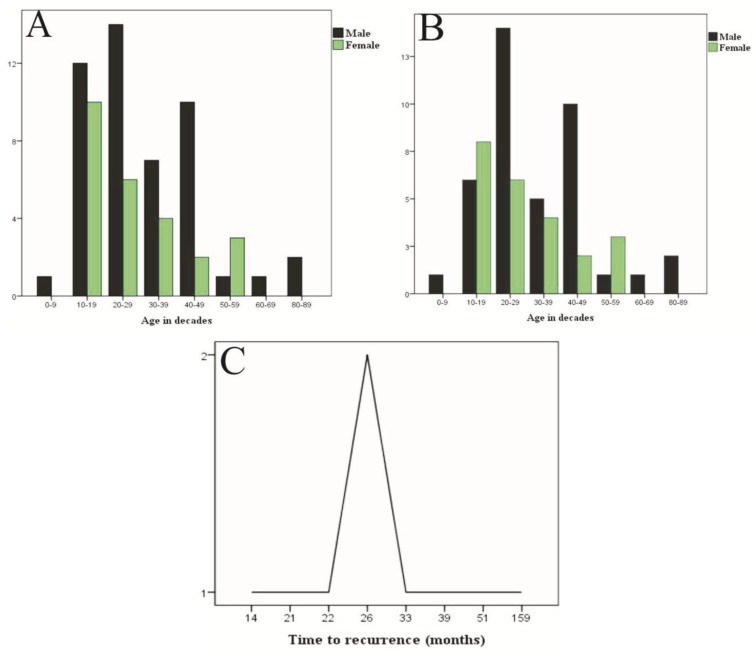
A, Age distribution of all patients with KCOT in decades. B, Age distribution of patients with solitary lesions. C, Simple line graph showing time taken from treatment of solitary KCOT to recurrence in months.

The profile of all patients can be found in  Table 1. There were 25 females and 50 males (overall male-to-female ratio of 2:1) with a total of 95 primary KCOTs (65 solitary and 30 multiple lesions). The patients were aged between 5 and 82 years (mean 29.4 years). Figure 1A shows the age (in decades) and gender distribution. Peak incidences were in the second and third decades although there was also a smaller peak in males in the fifth decade not replicated among the female patients. The mandible (77%) was more commonly affected than the maxilla (23%). The posterior mandible accounted for over 52% of the total cases of KCOT. Painless swelling was the most common complaint at presentation (76%) while about 10% were accidentally discovered by radiography. Impacted teeth were found to be related to 38% of total cases presented.

**Table 1:**
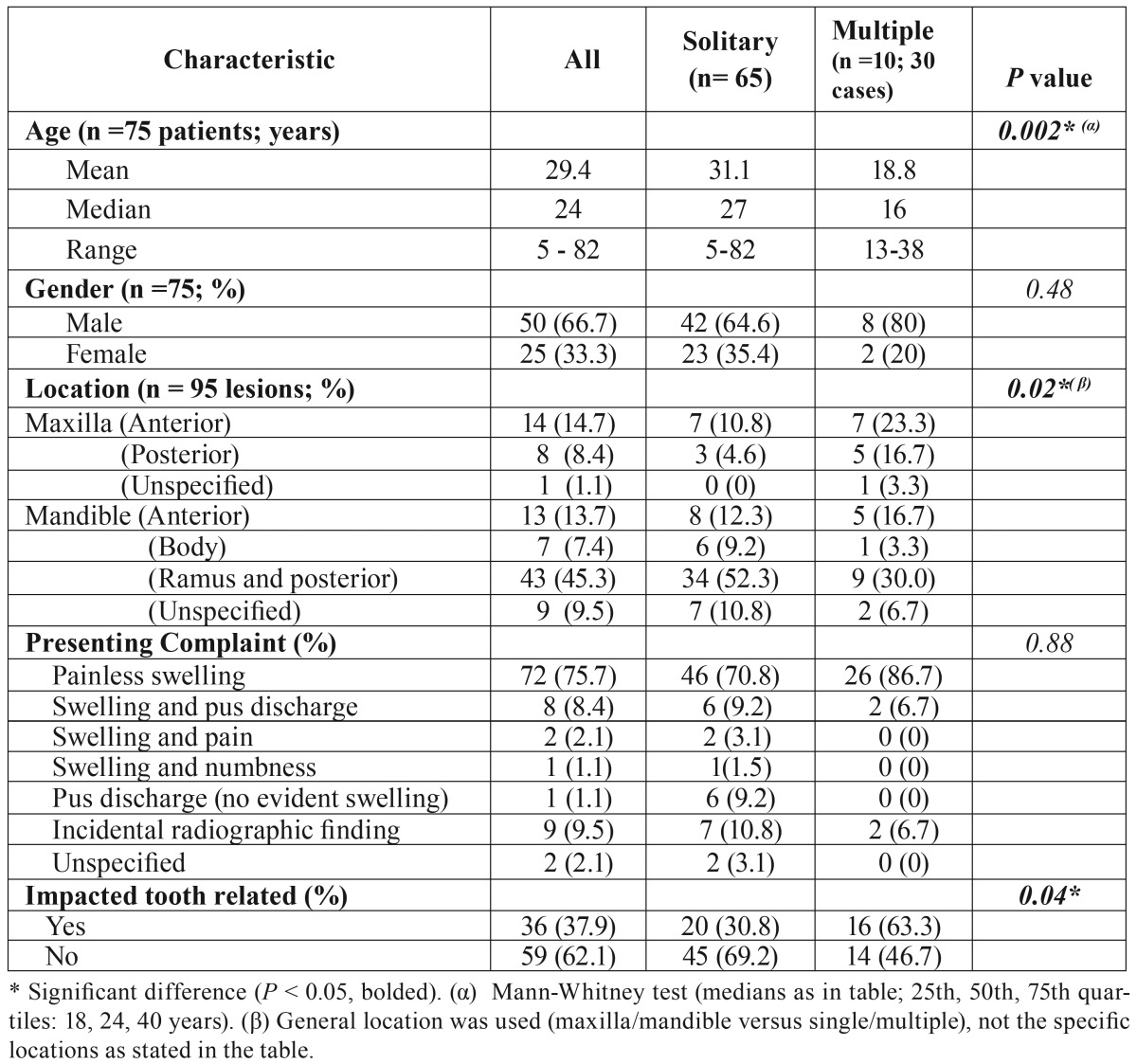
Demographic and clinical characteristics of 95 cases of KCOT in 75 patients.

**Figure 2:**
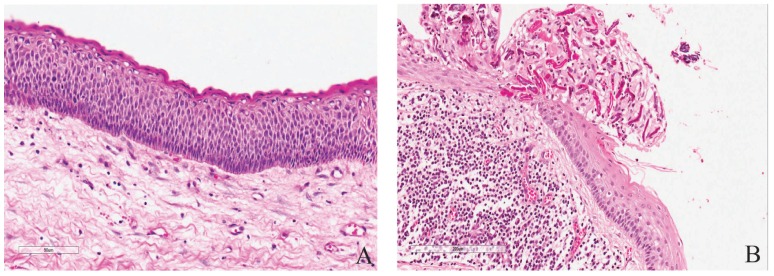
A, Classical features of KCOT with corrugated, parakeratinized epithelial lining, prominent, hyperchromatic and polarized basal layer and flattened epithelial-connective tissue interface. B, Inflammation-induced distortion of the epithelial lining with loss of parakeratinization, and prominent basal layer (left side), inflammatory exocytosis and Rushton hyaline body aggregates. The connective tissue is densely infiltrated by inflammatory cells. Scale bars: 90 µm and 200 µm respectively. Hematoxylin-eosin staining.

**Figure 3:**
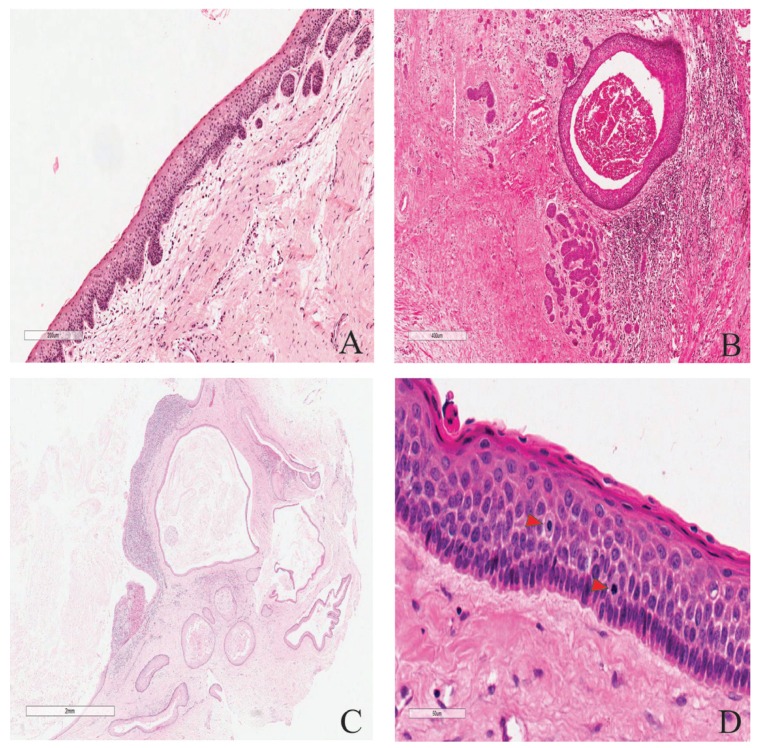
A, Basal cell budding disrupting the usual flattened lining-wall interface. B, Proliferating odontogenic epithelium in the wall of KCOT and cystification in a 38 year old male patient with NBCCS. C, Different cystic configurations in the satellite cysts of a 15-year old female with pitting of the palms. D, Two mitotic figures not far apart (40 x magnification) in a KCOT with high scoring suprabasal mitotic count (red arrows). Scale bars: 200µm, 400 µm, 2 mm and 50µm respectively. Hematoxylin-eosin staining.

Histologically, the general features of the cases were similar to those previously well described (1,2) (Fig. 2A). Some additional features were often seen. Intense inflammation adjacent the lining (or reaching it) may result in the lining looking similar to those of inflammatory odontogenic cysts and occasionally may have inflammatory exocytosis and Rushton hyaline bodies’ accumulation focally (Fig. 2B). The flattened epithelium-connective tissue interface may also be distorted by inflammation, or less commonly by basal cell budding (Fig. 3A). The connective tissue wall may harbor proliferating odontogenic epithelium (Fig. 3B), satellite cysts (Figs. 3B,3C) which may or may not acquire the full features of the main cyst, and inflammation-related changes such as cholesterol granulomas and dystrophic calcification.

- Solitary KCOTs

The gender distribution and the mean age of this group were similar to those of the general patient profile above. The peak age incidences for men were in the 3rd and 5th decades while those for the women were in the 2nd and 3rd decades (Fig. 1B). The mandible accounted for 86% (with the posterior mandible more than 62%) of total cases ( Table 1). There were significant differences between this group and the multiple KCOTs regarding some histological parameters such as mitotic count (Fig. 3D), satellite cysts and proliferating odontogenic epithelium ( Table 2).

**Table 2:**
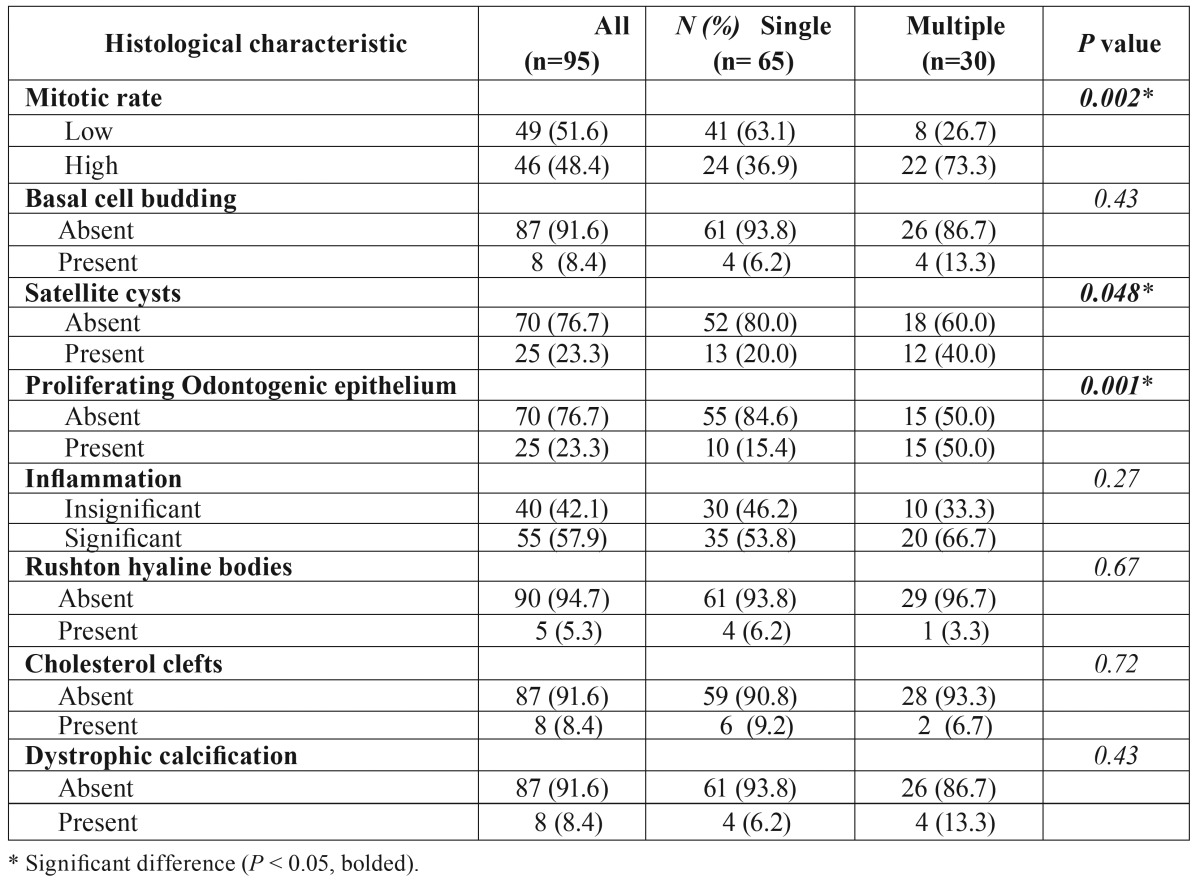
Histological features of 95 cases of KCOT in 75 patients.

- Multiple KCOTs

The profile of the patients in this group in our laboratory records is shown in  Table 3. Only one patient from this group previously had a formal diagnosis of NBCCS. Based on current diagnostic criteria suggested by the First International Colloquium on NBCCS (12), two previously undiagnosed female patients were also considered as having NBCCS because they were less than 20 years old at the time of presentation and had at least an additional major feature ( Table 3, footnote). No record of their genetic testing or molecular confirmation was available. The male-to-female ratio was 4:1 in this group. The mean age at presentation (18.8 years) was significantly less than that for single cases. All but 2 patients were in their second decade of life. There was a significant difference in the jaw site presentation (more maxillary lesions) in this group when compared with the solitary cases. Similarly, association with impacted teeth was significantly higher ( Table 1). The lesions in this group were often associated with significantly higher mitotic count, satellite cysts and proliferating odontogenic epithelium in the connective tissue wall ( Table 1).

**Table 3:**
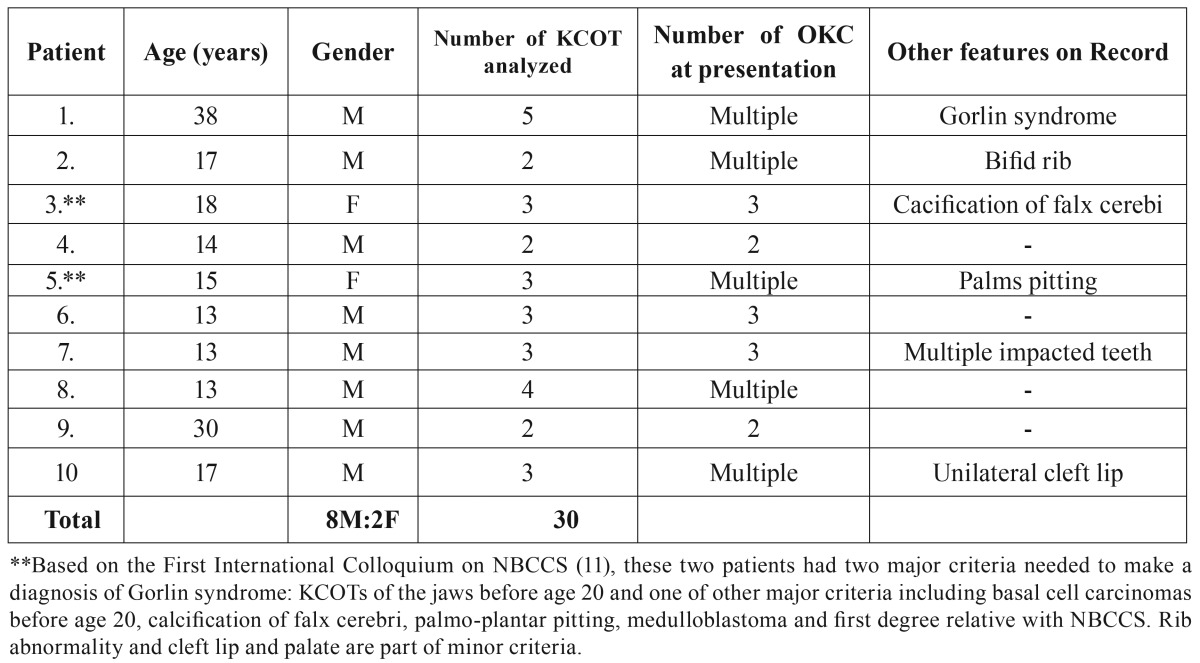
KCOT patients with multiple lesions.

- Recurrent KCOTs

All nine cases of recurrence were found in the mandible: 8 were in the posterior mandible (7 in the ramus/angle area) and one in the anterior mandible. The median time to recurrence was 26 months (range 14-159 months; Fig. 1C). Primaries that later recurred were initially compared with the solitary cases, and thereafter with 9 clinically-matched (by age, gender and location) patients with solitary lesions. There was no statistical difference in either the clinical or histological parameters for these cases (data not shown). Further comparisons were then made with the multiple cases ( Table 4). The age, the location and presence of odontogenic epithelium were found to be significantly different when primaries that later recurred were compared with the multiple cases. When the actual recurrent KCOTs were compared with multiple KCOT mitotic count and proliferating odontogenic epithelium were found to be significantly different between them.

**Table 4:**
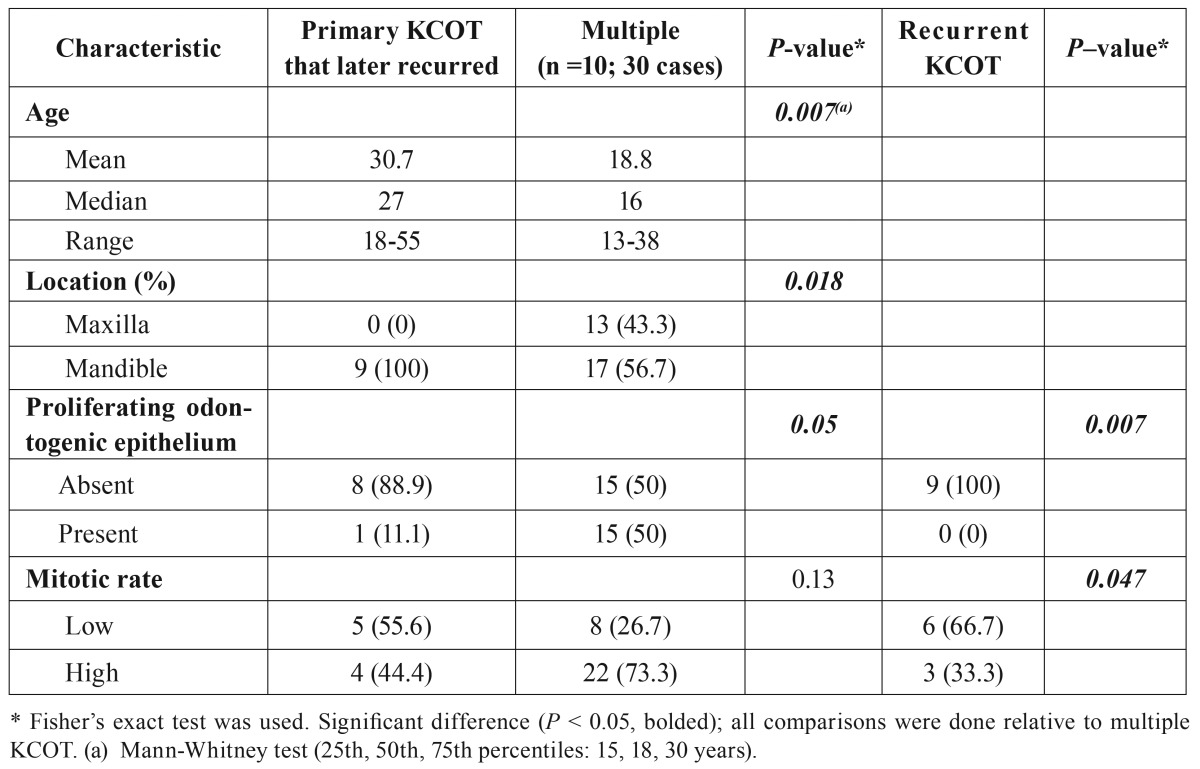
Clinicopathologic characteristics that were different between recurrent and multiple cases of KCOT in Saudi Arabian patients.

## Discussion

Since 1956 when it was first described, the odontogenic keratocyst has been classified as a jaw cyst although several workers have over the years raised the possibility that it should be considered a tumor (6). Although the World Health Organization (WHO) reclassified it as KCOT in 2005, several workers have continuously disagreed with this reclassification (13). It is an intraosseous odontogenic lesion with a fairly distinctive histological appearance and clinicopathologic characteristics. The cells of origin are believed to be from the dental lamina rest (rest of Serres).

Regarding the gender susceptibility, it is almost a consensus in the literature that KCOT is more common in males (14,15). This study found a relatively stronger male predilection than in some studies (14) but similar to others (15). A rare female predominance has been reported in Brazilians (16). In the subset with multiple lesions, the strong male predilection in Saudi Arabian patients (male-to-female ratio of 4 to 1) is in contrast to some studies that suggested female predilection (5,17). However, a review of several studies on NBCCS suggested a relatively equal gender prevalence (18). This discrepancy is most likely due to incomplete confirmation of the nature of the multiple lesions in the present study. In this study, at least one male and two female patients were considered to have NBCCS.

The mean age of presentation and the general age distribution are similar to those in the literature. Most patients seemed to be diagnosed in the second and third decades (16). Although varying explanations have been previously given for these peak incidences, this study aligns with that suggesting that patients with multiple OKCT tend to cluster in these two decades at initial presentation with most of the remaining cases diagnosed in the 4th decade. This may have substantial contribution to creating these peaks. Some studies had peaks in the 3rd and 4th decades, most especially the 3rd decade (14,15). This was also the peak age incidence in this study for patients with solitary lesions. The additional finding of a third peak for male patients with solitary lesions in the 5th decade is relatively not common across many studies although previous documentation (gender preference unknown) has been made in some (19,20). The mean age of patients with multiple lesions was found to be significantly less than the mean age of patients with solitary lesions. The simple explanation for this is that most patients with multiple (and indeed NBCCS-related) KCOTs present in their first three decades of life while solitary lesions appear to be well capable of occurring at any age.

The least disputed clinical feature of OKCT across virtually all studies is the location where it most commonly occurs. The mandible remains the choice location for KCOT, here accounting for about 77% of cases. This is compatible with prevalence ranging from 65% to 83% (21). Most lesions were located in the posterior aspect of the mandible in similarity with previous studies (14,15). It is interesting that a substantial percentage of the cases occurring in the maxilla were contributed by patients with multiple lesions resulting in a significant difference in comparison to solitary KCOT. Multiple KCOTs (particularly NBCSS-related) tend to be less discriminatory with location than sporadic cases. Multiple lesions simply affect multiple sites in the jaws in most cases.

The presenting complaints in KCOT patients were not significantly different whether they had solitary or multiple lesions. Interestingly, a subset of patients in either group had no complaint but their lesions were incidentally discovered on radiographic examination (9.5%). KCOT is symptomatic in about 50 - 90% of cases with the majority complaining of painless swelling or swelling in association to pain or purulent discharge (21). This is compatible with this study. Association of KCOT with impacted teeth is said to be in the range of 25-40% (22). Unlike dentigerous cysts where its development actively causes impaction by pressure of the cystic fluid on the tooth, impaction in KCOT is presumably a passive collateral event secondary to its growth. The significantly higher rate of teeth impaction in multiple cases as demonstrated in this study is testimonial to its higher growth potential than solitary lesions.

In this study, of all the histological variables evaluated, suprabasal mitoses in the epithelial lining, proliferating odontogenic epithelium and satellite cysts in the wall of KCOT were significantly more common in the multiple cases than solitary. These findings are similar to those of other workers (5). They are indicators of higher proliferative capacity in KCOT. The general observation is that these three parameters are also more numerous in the individual multiple cases in comparison to the individual solitary lesions. Each proliferating odontogenic epithelium in multiple lesions also tended to be smaller and extend to longer distances than those of solitary lesions. The satellite cysts in both types usually take 3 forms: rounded keratin-filled cyst lined by flattened or cuboidal cells which can attain a very big size; squamoid structures with central degeneration occupied by epithelial debris; and small irregular shaped cysts with lining indistinguishable from that of the main cyst (Fig. 3C). The multiple KCOTs with satellite cysts have a tendency to be populated more by the latter two while the solitary KCOTs have more of the first two types. It has often been postulated that basal cell budding may be important in the formation of satellite cysts (23,24). Based on this study, and in line with others (5), this phenomenon may be overrated. The histological evidence is more in support of satellite cysts arising from the proliferating epithelial rests of Serres (5).

This study embarked on reevaluating the solitary KCOTs that later recurred specifically to find out if they have features of high proliferative activity. The latter has often been part of the discussion (alongside basal cell budding and treatment methods employed) when the relatively high recurrence rate of KCOT is being considered (1,25). Comparison of solitary KCOTs that resulted in recurrence with clinically-matched, as well as all solitary lesions did not show any difference in their histological characteristics in agreement with previous workers (10). The only patient characteristic that seemed to be associated with recurrence was the location of the tumor. All the recurrent KCOTs were located in the mandible. Previous studies have documented preference of the mandible by recurrent KCOT (14,26). In comparing lesions that later recurred with multiple KCOT, the finding of no significant difference in the mitotic count between the two lesions is an indication that high mitotic count may be associated with recurrence although this should be taken with skepticism based on the few cases available for analysis. Proliferative index measured by Ki-67, a measure of active cells in the cell cycle has been shown to be associated with recurrence (27). The significant difference found between actual recurrent lesions and multiple KCOT in some indicators of higher proliferative activity implies the former may not be as aggressive as their original lesions that resulted in them. This suggests that they are probably not direct descendants of the main cyst but developed from proliferating odontogenic epithelial rests in the wall left during surgery.

Traditionally, surgeons and pathologists have usually attributed recurrence to the activity of satellite cysts formed as a result of basal cell budding and their detachment into the connective tissue and subsequent cystification (24,28) or to formation of satellite cysts from the proliferating epithelial rests within the cyst wall (5). At present, the most plausible explanation remains that recurrence occurs as a combination of treatment method employed as the major factor, with the histological factors playing a minor role. If the cyst is completely removed without leaving any of its content behind, chances of recurrence is low, but still present. Woolgar *et al.* (10) suggested that recurrence may be due to 3 factors: incomplete removal of the KCOT, development of new lesions from proliferating odontogenic rests or from satellite cysts left behind during surgical treatment and development of a completely new lesion in an adjacent part of the jaw that is then interpreted as a recurrence. This latter hypothesis may explain why it is difficult to differentiate recurrences from new lesions in patients with multiple lesions.

In conclusion, this study has reviewed the clinical and histological features of KCOT as seen from the perspective of a biopsy service in a Saudi Arabian population. The findings are similar to those of other parts of the world, suggesting that KCOT does not have racial discrimination in its expression. Recent works seemed to be directed to finding the molecular pathways of importance in the pathogenesis and clinical behavior of KCOT which will hopefully provide answers (which have not been provided by clinicopathologic studies) to the many questions regarding management of the lesion, whatever the form presented by the patient (29), in addition to a definitive statement on its nature either as a cyst or as a tumor (13).

Comment: The author adopted the term ‘keratocystic odontogenic tumor’ in line with the WHO. It is not implied that he agrees with its classification as a tumor. The word ‘lesion’ has been substituted for tumor in many other places in this work in place of KCOT.
